# Scaling the size of perimetric stimuli reduces variability and returns constant thresholds across the visual field

**DOI:** 10.1167/jov.21.11.2

**Published:** 2021-10-07

**Authors:** Phillip Bedggood, Selwyn Marc Prea, Yu Xiang George Kong, Algis J. Vingrys

**Affiliations:** 1 Department of Optometry & Vision Sciences, University of Melbourne, Parkville, Victoria, Australia; 2 Royal Victorian Eye and Ear Hospital, East Melbourne, Australia; 3 Centre for Eye Research Australia, East Melbourne, Australia

**Keywords:** automated perimetry, psychometric function, test-retest reliability, size

## Abstract

The conventional stimulus for standard automated perimetry is fixed in size, giving elevated contrast thresholds and reduced test reliability in the periphery. Here, we test the hypothesis that appropriate scaling of the size of perimetric stimuli will return fixed thresholds and reduced variability across the visual field. We derived frequency-of-seeing (FOS) curves in five healthy subjects at central (3 degrees) and peripheral (27 degrees) locations with a method of constant stimuli (MOCS) using a desktop LCD display. FOS curves for a Goldmann III (GIII) stimulus were compared with those for size scaled spots. To consider clinical translation, we tested a further five healthy subjects (22–24 years) with the Melbourne Rapid Fields (MRF) tablet perimeter at several locations spanning 1 degree to 25 degrees from fixation, deriving FOS curves (MOCS) and also conducting repeated adaptive clinical thresholding to assess intra- and interobserver variability. We found that GIII contrast thresholds were significantly elevated in the periphery compared with the parafovea, with concomitant reduction of FOS slope. Using appropriately size scaled spots, threshold and slope differences between these locations were significantly reduced. FOS data collected with the tablet perimeter confirmed that size scaling confers broad equivalence of the shape of the FOS curve across the visual field. Repeated adaptive thresholding with size scaled stimuli gave relatively constant intra-observer variability across the visual field, which compares favorably with published normative data obtained with the GIII stimulus. The reduced variability will improve signal-to-noise ratio for correct classification of normal visual field test results, whereas the lower contrast thresholds yield greater dynamic range, which should improve the ability to reliably monitor moderate defects.

## Introduction

Conventional perimetric testing uses a spot of fixed size equivalent to the Goldmann III target (GIII). However, implementation of the GIII spot size was never rigorously justified for perimetric testing, instead becoming a de facto standard due to its adoption by early commercial devices (Swanson, 2013). Despite its widespread use, the fixed size GIII spot might not provide an ideal test target given that the spatial tuning of cortical cells varies with eccentricity (Pan & Swanson, 2006). This mismatch may explain the poor retest variability generally observed in the periphery with conventional perimetry (Heijl, Lindgren, & Olsson, 1987). High retest variability is a fundamental limitation for clinical decision making, creating “noise” which can often mask the “signal” of functional loss due to disease (Artes & Chauhan, 2009).

Appropriate scaling of the size of perimetric stimuli with eccentricity should provide better spatial tuning of retinal ganglion cell perceptive fields (Khuu & Kalloniatis, 2015; Redmond, Garway-Heath, Zlatkova, & Anderson, 2010; Zele, O'Loughlin, Guymer, & Vingrys, 2006), as well as returning thresholds that do not vary with eccentricity (Zele et al., 2006). It is this latter approach that we will consider in this manuscript because it is well known that threshold variability shows an inverse relationship with threshold (Chauhan, Tompkins, LeBlanc, & McCormick, 1993; Donner, 1992; Henson, Chaudry, Artes, Faragher, & Ansons, 2000; Weber & Rau, 1992), implying that constant thresholds should be accompanied by constant variability. With a judiciously chosen scale, it should be possible to improve variability in the periphery compared to that obtained with the GIII stimulus.

Several studies have considered the relationship between stimulus size and threshold across the visual field, providing evidence that variation in spot size with eccentricity can “correct” for variations in sensitivity with eccentricity and so equate thresholds between central and peripheral locations (Latham, Whitakerxy, & Wild, 1994; Sloan, 1961; Vingrys, Healey, Liew, Saharinen, Tran, Wu, & Kong, 2016; Wilson, 1970). Sloan's (1961) data establishes the concept that stimulus size can be considered as a test variable in much the same way that intensity is conventionally modulated at a fixed stimulus size in a traditional perimetric task (Sloan, 1961). Wilson (1970) extended Sloan's observations with finer gradations in stimulus size (Wilson, 1970), establishing that spot diameter can be scaled to return fixed thresholds across eccentricities ranging from 5 degrees to 50 degrees in her two subjects. The findings of Wilson are useful if trying to define a scale to give constant thresholds across the visual field, although the background luminance (212 cd/m^2^) used was greater than that in traditional perimeters (Wilson, 1970), which leaves unclear the relevance of this work to standard automated perimetry.

Latham et al. (1994) formally tested the spot scaling theory in 20 normal observers (10 young, mean age 24 years; and 10 elderly, mean age 72 years) from 6 degrees to 30 degrees eccentricity along four meridia using a standard perimetric task (Humphrey Visual Field Analyser) and six Goldmann targets (G0 to GV; Latham et al., 1994). Their data indicate that size scaling produces diminishing returns in both age groups that suggest little further utility in increasing stimulus size beyond about a Goldmann size V target (1.72 degrees diameter). Similar diminishing returns behavior was established in the fovea by Zele et al. both in patients with age-related macular degeneration and in healthy controls; little further utility is evident beyond stimulus diameters of about 1.6 degrees (Zele et al., 2006). Latham et al. proposed that age produces a loss of sensitivity that can be redressed by size scaling (Latham et al., 1994), which is also supported by the data of Zele et al. who show that beyond around 1.6 degrees (very large spots) a residual 1.2 dB depression remains in the foveal thresholds of older eyes. More importantly, the data of Latham et al. (1994) collapse onto a common template as a function of size in both age groups, which indicates that appropriate size scaling up to 1.6 degrees should return fixed thresholds at all eccentricities out to 30 degrees in both young and older observers (Latham et al., 1994). These considerations appear to hold in glaucoma, where it has been shown that test-retest variability is similar or better with size V stimuli (Wall, Doyle, Zamba, Artes, & Johnson, 2013), but slightly worse for size VI stimuli (Wall, Doyle, Eden, Zamba, & Johnson, 2013). The area of complete spatial summation is generally less than a size V stimulus, despite a general increase in spatial summation with glaucomatous damage (Redmond et al., 2010).

The more recent work of Kuu and Kalloniatis, who investigated spatial summation across the visual field, concurs with the finding of Latham et al., showing by interpolation that an appropriately scaled spot can provide a constant threshold across all regions of the visual field (Khuu & Kalloniatis, 2015). Similar findings have been reported for more complex stimuli (Keltgen & Swanson, 2012). The effect of spot scaling for perimetry has also been demonstrated with the Melbourne Rapid Fields (MRF) perimeter, which applies an interpolated scale from Sloan's data (Sloan, 1961) to scale perimetric targets in order to equate thresholds across the visual field out to 30 degrees (see Figure 5, Vingrys et al., 2016).

The scale suggested by the above studies results in larger spots at peripheral locations than a GIII stimulus, with the proviso that the maximum size be about a diameter of 1.72 degrees (approximately Goldmann size V, see reasoning above). Although the above studies show that such scaling can produce constant thresholds (Latham et al., 1994; Sloan, 1961; Vingrys et al., 2016; Wilson, 1970), the effect that this has on threshold variability for simple spot detection perimetry is not clear. There is reasonable expectation that larger spots should both tighten variability and lower threshold in the periphery, because threshold and variability (inversely related to the slope of the psychometric function) are directly related and generally in proportion to one another (Chauhan et al., 1993; Donner, 1992; Henson et al., 2000; Weber & Rau, 1992). This means that not only might one be able to lower threshold with size scaled spots, but also to reduce variability with the same scaling. In essence, this should yield a better signal-to-noise ratio (SNR) for making clinical decisions regarding the normality of patient results.

The above reasoning may explain the findings of clinical studies showing that stimuli larger than GIII can improve detection of glaucomatous loss (Rountree, Mulholland, Anderson, Garway-Heath, Morgan, & Redmond, 2018; Swanson & King, 2019; Wall, Doyle, Eden, et al., 2013). Such findings have been accompanied by suggestions that variable spot size (instead of, or in addition to, luminance) should be used in determining visual thresholds. This concept first emerged with “ring” perimetry, which varies the size of a high pass annulus target that is equiluminant with the background (Frisén, 1993). Ring perimetry was found to produce lower test-retest variability than conventional perimetry in healthy subjects, and may also improve the detection of glaucomatous progression (Chauhan, House, McCormick, & LeBlanc, 1999). Similar benefits to modulating stimulus size, rather than contrast, have been reported for conventional detection tasks as well (Rountree et al., 2018; Wall, Doyle, Eden, et al., 2013). These benefits may result from an enlarged area of spatial summation in glaucoma and underscore the importance of spot size as a perimetric variable (Frisén, 1993; Redmond et al., 2010). In addition, the MRF perimeter, which uses a size scaled spot, as described above, has been shown to yield a smaller coefficient of repeatability in clinical trials to that returned by the Humphrey Field Analyser (HFA) 24-2 test (SITA standard), which uses a GIII spot (MRF = 5.7 dB vs. HFA = 7.9 dB; Prea, Kong, Mehta, He, Crowston, Gupta, Martin, & Vingrys, 2018). Improved test-retest variability has also been reported with the use of sinusoidal stimuli that are appropriately scaled in size with eccentricity to equate threshold (Swanson, Horner, Dul, & Malinovsky, 2014).

The above studies support but do not directly test the hypothesis that appropriately size scaled spots will equate and improve the slope of the psychometric function across the visual field. If this were shown to be so, using larger spots in the periphery would be accompanied by a decrease in threshold variability, as evident in the data of Rountree et al. at a single eccentricity (Rountree et al., 2018). The reduced variability would allow a given level of accuracy to be reached in fewer trials or for test accuracy to be improved given a fixed number of trials. Both elements are desirable outcomes for clinical perimetry.

Given the hypothesis that increasing spot size improves peripheral threshold variability has never been rigorously or directly tested, we aimed to compare thresholds and threshold variability of both GIII stimuli and size scaled spots across the visual field. We used an MOCS procedure to provide a rigorous test of the hypothesis, alongside routine perimetric threshold testing on the MRF to establish clinical relevance.

## Methods

### Overview

Two studies were conducted, each using normal subjects with healthy eyes. The first used an MOCS generated on a desktop LCD screen using size scaled spots and GIII targets at eccentricities representative of central and peripheral perimetric locations (*x* and *y*: 3.3 degrees and 27.3 degrees). The second used the MRF tablet perimeter (Glance Optical Pty Ltd, https://www.visioninhome.com/), which makes use of a fixed spot size scaling with eccentricity, to establish the clinical relevance of the findings and to confirm constancy of threshold and threshold variability with eccentricity: this was assessed by both an adaptive Bayesian thresholding method and a method of constant stimuli (MOCS).

### Subjects

Testing procedures and informed consent complied with the tenets of the Declaration of Helsinki and were approved by the University of Melbourne, Human Research Ethics Committee (#1749228).

Ten healthy subjects were recruited from students and staff at the Department of Optometry and Vision Sciences, The University of Melbourne, Australia. Five staff participated in the LCD study (4 men and 1 woman), ranging in age from 32 to 68 years (including 3 of the co-authors). Five perimetrically naïve students participated in the MRF study (4 women and 1 man), ranging in age from 22 to 24 years.

All subjects had best corrected visual acuity of 6/6 or better at distance in the eye under test (right eye), and wore their habitual near correction if needed. Spherical refractive error ranged from +1.25 to −6.50 D in the LCD study, and from −0.25 to −3.50 D in the MRF study, with a maximum of −1.50 D astigmatism across both groups. All subjects had normal color vision on Ishihara plate testing. There was no pathology in any test eye.

### Spot size

Stimuli were either fixed in size to match the GIII stimulus (diameter = 0.43 degrees) or were scaled in size (diameter) with eccentricity. The diameters of size scaled spots were interpolated from iso-threshold values reported by Sloan (Sloan, 1961). These values were further scaled to lie between Goldmann size II and Goldmann size V, as described elsewhere (Vingrys et al., 2016) in order to ameliorate the effect of refractive error on test spots. An additional scaling was applied to account for the non-curved nature of the displays (tangent perimeter). The formula used to encompass the above considerations and generate our stimuli was:

$$d=0.00271\;E^{1.714}+0.226$$

where d is the diameter of the spot in degrees, and E the eccentricity from fixation in degrees. The overall scaling yields a GIII sized spot at approximately 12 degrees eccentricity, with smaller spots closer to the fovea (approximately size II at 1 degree) and larger spots in the periphery (approximately size IV at 24 degrees). The extra scaling factors are not expected to alter the threshold/slope relationship, as this has been found to be constant up to 1 log unit above the critical size (Wilson, 1970).

The spot scaling detailed above is similar to that used by the MRF iPad perimeter and was chosen to return constant thresholds across the visual field in that device (Vingrys et al., 2016). The spot scaling has the added advantage that deeper peripheral defects may be better characterized compared with a GIII spot, as they are less likely to lie outside the dynamic range of the device (Swanson, 2013; Swanson & King, 2019).

### Display characteristics and procedures

#### LCD display

**Figure 1.**
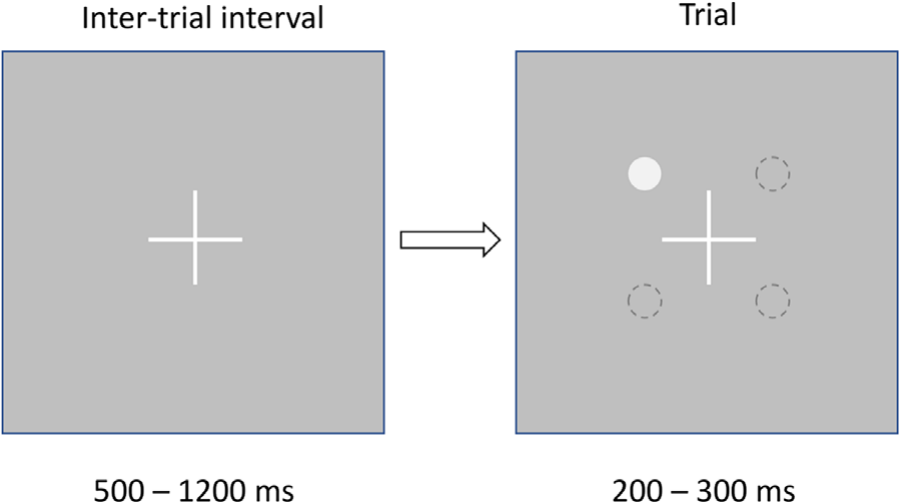
Schematic illustration of stimulus display. Fixation was maintained with a small cross for a minimum of (500–1200) ms, before displaying the stimulus at the test eccentricity in one of four quadrants equidistant from fixation and symmetric about the horizontal midline. Stimuli were displayed for 200 ms (LCD) or 300 ms (MRF). Stimulus display was accompanied by a brief tone in the LCD study, and subjects pressed a button for either “yes” or “no” to indicate whether they had seen the stimulus. In the MRF study, no tone was used and subjects tapped the tablet screen whenever a spot had been seen.

A Samsung SyncMaster 2243BW was driven by DVI-I output from an NVIDIA GTX 780 graphics card through a VGA adapter to achieve 9-bit luminance resolution. Luminance levels were gamma corrected by calibration with a PR650 spectro-photometer (Photo Research, Chatsworth, CA, USA). The frame rate of the display was 60 Hz and its maximum luminance was 63 cd/m^2^. Stimuli were displayed as luminous increments of 200 ms (12 frames) duration on a background of 20 cd/m^2^. A slightly brighter background was adopted compared with the 10 cd/m^2^ used by popular perimeters in order to generate smaller increments in contrast on the LCD display (at the expense of a reduced range of contrast able to be generated). This was necessary to obtain sufficient sampling of the slope of the psychometric function at the parafoveal test location. A yes/no testing procedure with auditory prompt was used to determine hit rate (Figure 1).

The LCD display generated both size scaled and GIII spots at the parafovea and periphery with spot locations chosen to coincide with the HFA 24-2 test grid along its horizontal locus (x = ±3 degree, y = ±3 degrees, and x = ±27 degrees, y = ±3 degrees). These locations will be identified as 3 degrees and 27 degrees throughout this manuscript although the Euclidean distance is in fact greater (4.2 degrees and 27.2 degrees). Stimuli at each eccentricity were presented randomly in one of four quadrants, positioned symmetrically about the horizontal line running through fixation (Jäkel & Wichmann, 2006), removing any incentive for subjects to shift fixation. One eccentricity (parafoveal or peripheral) was tested per run.

The MOCS paradigm implemented seven test intensities with 40 presentations at each intensity (10 in each quadrant) and hit rates were pooled across the 40 presentations for each intensity. An initial QUEST staircase estimated threshold and slope at a given location and this outcome seeded the MOCS run. Our goal was to achieve five steps on the slope of the FOS transition between the 10% and 90% hit rate: the other two test levels were estimated at plus or minus three standard deviations (SDs) either side of the mean, being easily seen or not seen. If the five levels on the FOS transition were not achieved (about 20% of runs), another MOCS was undertaken using an adjusted series of five optimally spaced luminance steps derived from the previous run. Testing required about 10 minutes per MOCS with rest breaks as needed. A cumulative Gaussian was used to model the frequency-of-seeing (FOS) curve from the measured hit rates, returning a mean (threshold) and SD (slope) after allowing for false negative and false positive responses derived from the average hit rates of all supra- or infra-threshold data (±3 SD) for that person. The false response rates ranged from 1.3% to 7.5% (average of 2.5%) between trials and we applied the average value (2.5%) for all modeling; we found that this did not bias curve fitting in any appreciable way.

#### MRF perimetry

**Figure 2.**
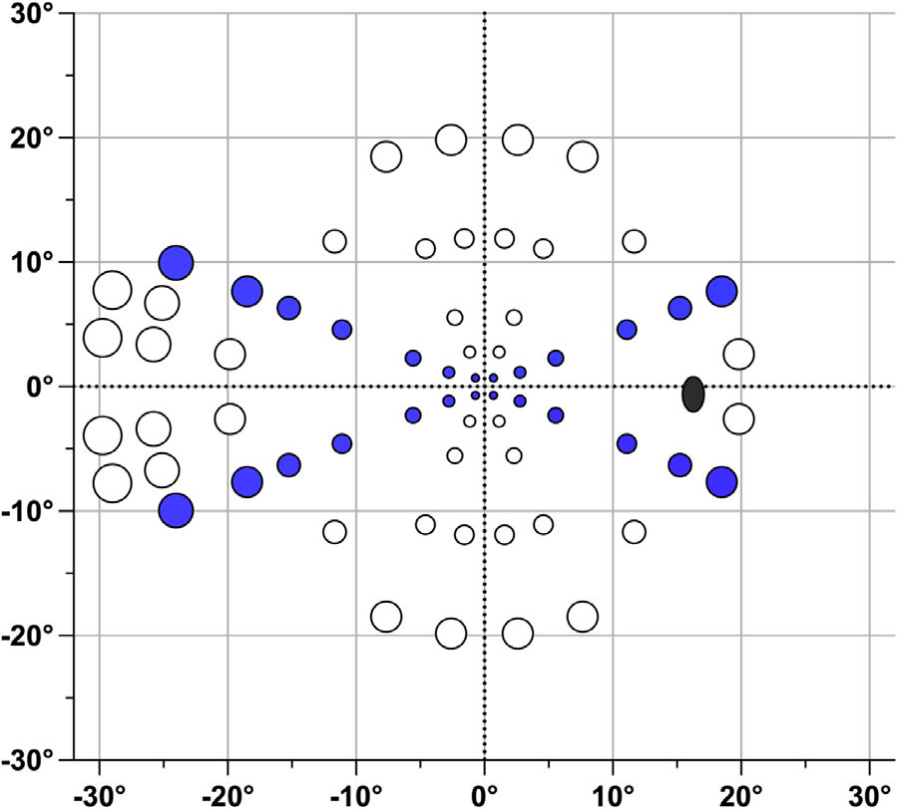
The MRF perimeter uses a test grid of 66 locations over the central 30 degrees × 20 degrees arranged in a radial pattern (right eye). Thresholds were repeated 8 times to return an average and standard deviation of threshold at the six or seven eccentricities located along the 22 degrees meridian either side of the horizontal (filled blue spots). An FOS curve was also determined at each eccentricity for the size scaled spots (see Methods). Variable spot size is shown for visualization and is not to scale: the black symbol shows the approximate location of the blind spot.

To increase the translational relevance of our findings, we used a commercial perimetry application running on an iPad tablet to test both a MOCS, at a limited number of locations, and an adaptive Bayesian threshold at 66 locations oriented in a radial pattern across the central visual field (Figure 2). All testing was undertaken at 33 cm with a chin and forehead rest to stabilize the head position. The iPad was gamma corrected and calibrated with an IL1700 radiometer and photopic filter (SED033), as detailed by Vingrys et al. (Vingrys et al., 2016). Each stimulus was displayed for 300 ms; this is a longer duration than the 200 ms of the HFA, but remains longer than saccadic “reaction time” for low contrast, peripheral stimuli (Warren, Thurtell, Carroll, & Wall, 2013). Stimuli were presented on a 5 cd/m^2^ background and thresholds were returned from a yes/no Bayesian estimate (Vingrys et al., 2016). This is similar to the approach used by SITA except that here the Bayesian logic is also used to drive spot brightness after each presentation unlike the staircase used by SITA. A single visual field test required about 4 to 5 minutes and was repeated eight times (with breaks as needed) to return an average threshold and SD for each eccentricity (average of 2 or 4 locations along the 22 degrees meridian; see Figure 2). Perimetry testing was undertaken with size scaled (Kong, He, Crowston, & Vingrys, 2016) spots with false-positive and false-negative assays interspersed throughout the test. Although thresholds and SD were established across the entire visual field, only those obtained from spots lying along the 22 degrees meridian (see Figure 2) were analyzed and compared to the FOS curves measured by an MOCS at these same locations, again using size scaled spots.

**Figure 3.**
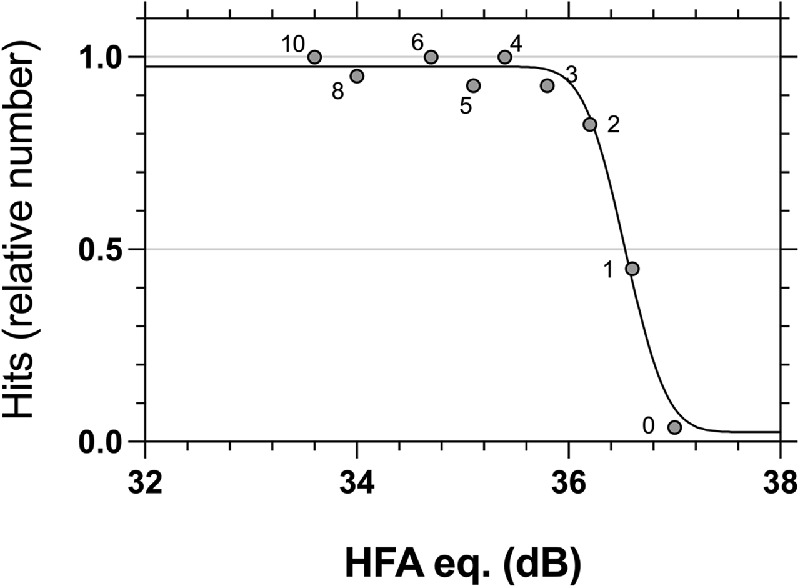
An example of a FOS measured on an iPad at 3 degrees eccentricity (grey circles show average group threshold) using nine discrete luminance levels, each +1 or +2 Digital Driving Levels (numerics, 1 DDL to approximately 0.35 dB) from its neighbor. Each DDL was averaged over 10 repeats in four quadrants (40 presentations and 5 participants), so each point has been derived from 200 data (in total) for each DDL at a given eccentricity. As it turns out, the 8-bit tablet under-sampled the transition of the MOCS yielding steeper slopes due to the undersampling.

The FOS on the MRF was determined at the locations shown in Figure 2 from the average hit rates of nine test intensity levels (including background) for size scaled targets. Intensity levels were designed to range from the background (for false positive estimates) to about 3 dB brighter, using incremental integer steps of the 8-bit digital driving levels (DDLs) from the background (Figure 3). DDL spacing was determined from pilot runs to span a range needed to ensure easy visibility of all targets. Spots at a given eccentricity were tested in the same test run. Intensity levels were shown in a random order during any run and each intensity was repeated 10 times per quadrant (40 per eccentricity) to establish an eccentricity related hit rate, as detailed above for the LCD display. A cumulative Gaussian was again used to model the average FOS curve at each eccentricity. Each MOCS session took approximately 1 hour, with breaks taken by the observer as needed.

### Subject input

For the LCD screen, each stimulus presentation was preceded by a tone at stimulus onset and the subject's task was to respond to the presence of a spot using a Thrustmaster Firestorm Digital 2 gamepad controller. An untimed response window was applied after spot presentation with subjects choosing one button for “yes” and another for “no.” False positive and false negative responses were polled from the extreme luminance targets.

With the MRF, subjects tapped the tablet screen at a designated touch zone when they thought that they had seen a spot in either the perimeter mode or during the MOCS. False positive and false negative responses were polled from the extreme luminance targets in the MOCS and using traditional perimetric sampling for visual field testing (Kong et al., 2016).

### Control of fixation

A small cross was maintained at fixation for both devices. To ensure fixation accuracy, the MRF uses a blind spot monitor during the perimetry test. Here, it was established that fixation was accurate with loss estimates ranging between 2.1% and 9.4% across all tests of all observers (mean = 4.2 ± 1.1%).

### Statistics

For ease of comparison with the perimetry literature, thresholds have been expressed in Humphrey equivalent (HFA_eq_) dB using the calibrated luminance of the tablet and desktop PC:

(1) $$HFA_{eq}=-10\;log_{10}\left(W.\frac{10}{3183}\right)$$

where W is the Weber contrast ratio (L_S_ - L_B_)/L_B_, with Ls the spot luminance and L_B_ the background luminance. The number 10 corresponds to the background luminance of the HFA and the number 3183 to the maximal HFA spot luminance, in cd/m^2^. Therefore, to convert the reported values in HFA_eq_ to the Weber contrast ratio W (as shown for example in the Table 1), one may use the following equation:

(2) $$W=\frac{3183}{10}10^{-HFA_{eq}/10}.$$

**Table 1.** Optimized curve fit parameters for the smooth lines shown in Figure 4.

	GIII spot		Size-scaled spot	
**FOS parameters**	3 degrees	27 degrees	3 degrees	27 degrees
Mean (HFA_eq_ dB)	37.5	32.1	36.1	35.3
Std. Dev. (HFA_eq_ dB)	1.1	2.4	1.2	1.6
Mean (log10 Weber)	−1.25	−0.71	−1.11	−1.03
Std. Dev. (log10 Weber)	0.11	0.24	0.12	0.16
**95% CI**				
Mean (HFA_eq_ dB)	34.4–34.6	28.9–29.3	32.9–33.3	32.2–32.7
Std. Dev. (HFA_eq_ dB)	0.95–1.23	2.04–2.71	1.07–1.29	1.37–1.85

CI, confidence interval; Std. Dev., standard deviation.

**Figure 4.**
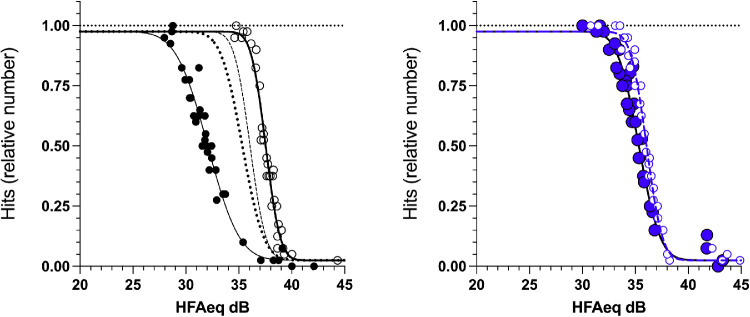
Average FOS at 3 degrees and 27 degrees returned from five subjects using GIII and size scaled spots on a 20 cd/m^2^ background (LCD display). With the GIII stimulus (left panel), threshold becomes elevated and slope flattened in the periphery (filled symbols) compared to the fovea (unfilled symbols). With size scaled stimuli (right, spot size shown to approximate scaling), these differences are significantly ameliorated. The panel on the left shows the scaled FOS curves reproduced from the right panel for comparison, as dashed and dotted lines for 3 degrees and 27 degrees, respectively.

FOS curves have been described by a cumulative normal transition (1-Φ). For grouped data, psychometric functions have been fit to all data after thresholds were equalized between individuals by shifting curves along the x-axis (Bedggood, Ahmad, Chen, Lim, Maqsudi, & Metha, 2020). This “equalization” step is necessary to avoid underestimation of the slope, which is expected with threshold differences between sessions (or in this case, individuals; Wallis, Baker, Meese, & Georgeson, 2013). To achieve equalization, individual FOS curves were established and a threshold returned for each individual. Individual data were then adjusted for differences from the group mean and a global model optimized to this adjusted data (see Figure 4).

An alternate approach to the one just described would be to average the fitted parameters across individuals; in line with other reports (Wallis et al., 2013) we find this “fit-then-pool” option noisier, yielding greater uncertainty (especially in the slope). Instead, we opted for the aforementioned “pool-then-fit” approach to reconstruct the slope of the average individual.

## Results

### Shape of the psychometric function

**Figure 5.**
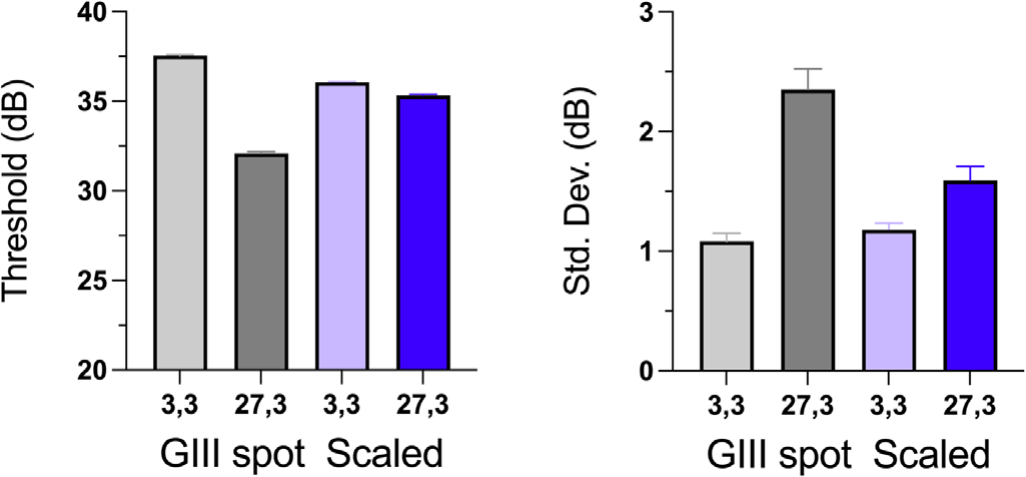
Optimized curve fit parameters for the cumulative gaussians producing the smooth lines of Figure 4. Error bars indicate 95% confidence interval for each parameter.

Figure 4 shows the FOS curves (hit rates) obtained at 3 degrees (open symbols) and 27 degrees (filled symbols) using the GIII stimulus (left) or size scaled stimuli (right), plotted as a function of spot brightness (HFA_eq_ dB). Individual data for the five participants have been equated for threshold differences and cumulative gaussian curves have been optimized to the resulting pooled data (solid lines). The outcomes and confidence intervals for these fits are also given in the Table 1 and Figure 5. The GIII data (left) confirm existing literature that there is a large drop in sensitivity between the center and periphery, which is accompanied by a decrease in slope of the psychometric function.

When spot size was scaled between the center and the periphery according to the data of Sloan (Sloan, 1961), such that spots are smaller in the fovea and larger in the periphery, threshold was elevated (i.e. lower HFA equivalent value, indicating reduced sensitivity) centrally but was lowered in the periphery as expected. However, the scaling adopted in our MOCS was not sufficient to equalize thresholds completely, with a difference of 0.8 dB (*p* < 0.05; see the Table 1) remaining between locations. However, this has reduced from the 5.4 dB difference found with the GIII stimulus.

The lowered peripheral thresholds were accompanied by a significantly steeper slope, corresponding to a significant drop in the fitted standard deviation of the cumulative normal, by 0.8 dB (from 2.4 to 1.6, see the Table 1). At the 3 degrees location, our procedure could not detect any reduction in slope as might be expected to accompany the small loss of sensitivity (1.4 dB; see the Table 1). Comparing foveal and peripheral locations, peripheral slopes remain significantly flatter than those of the 3 degrees location despite the size scaling (see the Table 1), again indicating that the scaling adopted was not sufficient to equalize performance between the two locations in these observers (although it was significantly improved).

**Figure 6.**
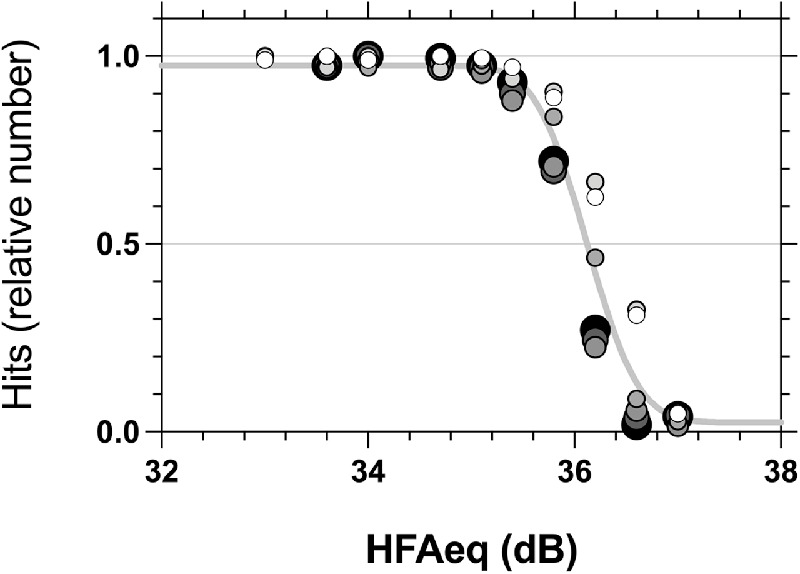
Average FOS across the visual field 1 degree, 3 degrees, 6 degrees, 12 degrees, 15 degrees, 18 degrees, and 25 degrees along the 22 degrees meridian returned from five subjects using size scaled spots on a 5 cd/m^2^ background (iPad display). Average FOS data are plotted for the size scaled stimuli at each eccentricity (grayscale symbols, larger symbols indicating greater distance from fixation). A single curve is fit to this data (solid grey line) to indicate that there is little difference in performance across the visual field with this scaling.

We also show FOS data obtained by MOCS using the MRF tablet perimeter on a different group of five young, healthy subjects (Figure 6). Data were collected with size scaled stimuli at 1 degrees, 3 degrees, 6 degrees, 12 degrees, 15 degrees, 18 degrees, and 25 degrees eccentricity (colored symbols of Figure 1). As described in the Methods and shown in Figure 3, toward the fovea there was insufficient display contrast to accurately measure the slope of the psychometric function. For this reason, we do not attempt to draw statistical comparisons between fitted parameters as above, but instead present the raw data to demonstrate qualitative overlap of the psychometric functions between all eccentricities over the tested contrast range. This overlap is illustrated by a single cumulative normal optimized to the size scaled data from all eccentricities (solid grey line in Figure 6).

As noted above, the adopted size scaling does not completely account for eccentricity-dependent differences in performance. The foveal points (1 degrees and 3 degrees; orange and red symbols) appear to have higher thresholds by about 1.0 dB (data shifted to the right). However, these residual differences are small compared with the pronounced drop in performance for the GIII stimulus between the periphery and the 3 degrees location (outermost two curves), noted above with the desktop display (see Figure 4).

### Adaptive threshold estimates

**Figure 7.**
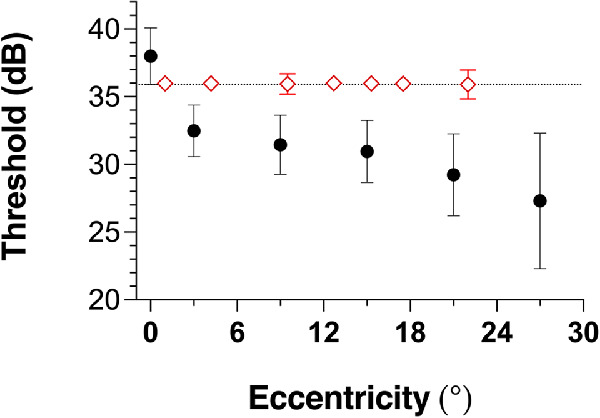
Mean and standard deviation in adaptive thresholds along the 22 degrees meridian for size scaled stimuli measured by the MRF (unfilled diamonds), together with data reproduced from the literature for the GIII stimulus at comparable eccentricity (Heijl et al., 1987) (filled circles). Left shows mean threshold (symbols) and interobserver standard deviation (error bars). Right shows the average intra-observer standard deviation.

To demonstrate clinical relevance, Figure 7 shows adaptive Bayesian threshold data collected with the MRF for the same group of young observers whose data was shown in Figure 6, again using size scaled spots but this time presented at locations corresponding to the 24-2 SITA testing pattern of the HFA. Tests were repeated eight times for each subject at each test location. Points near the blind spot were removed, as were outliers where threshold on a run was 20 dB or more below the median at a given location (3 of 1915 outliers removed). The remaining thresholds were pooled across all subjects, across repetitions, and across quadrants of the visual field. The figure plots the mean (open symbols) and standard deviation (error bars) in threshold estimates pooled in this way. For comparison are plotted normative data (*n* = 88) for a GIII stimulus from Heijl et al. (Heijl et al., 1987), with intrasubject variability sourced from their figure 3 (filled symbols). This is a conservative comparison because the variability in our data stems not just from test-retest variability, but also from between-individual and anatomic factors. However, it should be noted that our limited sample of young, laboratory-recruited subjects may be expected to show reduced variability in general compared with the randomly selected population sample undertaken by Heijl et al.

Figure 7 shows comparatively little decline in MRF threshold with eccentricity when the size scaled stimulus is used, as predicted by the FOS data of Figure 6. In contrast, the GIII stimulus has similar thresholds in the fovea but a 5.5 dB drop in threshold at 3 degrees after which thresholds reduce naturally with eccentricity out to 27 degrees. In the periphery, we find greater thresholds for the size scaled spot compared to published estimates for the GIII stimulus (Heijl et al., 1987), effectively yielding a larger dynamic range for testing. Variability was also smaller and relatively constant across the central visual field with size scaled spots, again consistent with Figure 6 based on the similar appearance of the FOS curves between most locations. The data reproduced for the GIII stimulus confirms the well-known decline in sensitivity with eccentricity, with concomitant increase in intra-observer variability, which we propose is due to the flatter FOS slope detailed above.

## Discussion

The “hill of vision” is a well-known phenomenon whereby perimetric sensitivity declines with increasing distance from fixation. Previous investigators have shown that a perimetric stimulus can be scaled in size to return fixed thresholds across the visual field such that the “hill” expected for an average subject will flatten out. Here, we propose that threshold variability should also be reduced due to the empirical relationship, observed in health and disease and across a number of visual functions, between threshold and variability (Chauhan et al., 1993; Donner, 1992; Henson et al., 2000; Weber & Rau, 1992), as well as between threshold and slope of the psychometric function (Rountree et al., 2018; Wall, Doyle, Eden, et al., 2013). Steeper psychometric functions result in reduced test-retest threshold measurements, especially for a limited number of trials as in conventional perimetric testing (Chauhan et al., 1993). Of course, it is possible that changes in neural wiring across the visual field could lead to differences in slope that are not directly tied to differences in threshold. In this study, we directly tested the hypothesis that size scaled spots will not only lower thresholds but also increase slope of the psychometric function across the visual field, and that the improved slope would result in low test/retest variability in a clinical test in the presence of a low number of trials.

In regard to size-related threshold effects, our findings echo those of previous investigators, where the use of a GIII spot in the periphery produced markedly elevated thresholds (lower HFA equivalent) than at the parafovea. Using the scaling implied by Sloan (Sloan, 1961) such that spot size was increased in the periphery and decreased centrally, our results show strong amelioration of threshold differences between the fovea and periphery. The proposed scaling, however, left residual threshold “error” of 0.8 dB between the central and peripheral locations for the average subject. Variability was commensurately higher (see the Table 1). It is possible that a different scaling could correct for these residual errors; we did not set out to confirm the precise scaling required in this study, but the issue warrants further investigation.

The largest change in sensitivity with eccentricity for a GIII spot appears over the foveal region, as evident in Figure 7 when comparing our data to that in the literature (Heijl et al., 1987). Although the data presented from Heijl et al. was collected in an older age group (20–80 years old), age does not explain the sudden drop in threshold as evident from the age-related relationship that they plot (see their figure 1). Indeed, Khuu and Kalloniatis (Khuu & Kalloniatis, 2015) show similar trends in a group of younger observers (median age 28 years: see their figure 2) that is a function of spot size. It should be noted that commercial perimeters typically measure stimuli at the fovea with a series of repeated presentations at fixation; the lack of uncertainty in stimulus location may partially explain the elevated sensitivity at the fovea. However, Khuu and Kalloniatis (2015) used the same testing strategy to compare different spot sizes, confirming that the foveal elevation in sensitivity is significantly ameliorated by the use of large spots (Goldmann size V). Both of these studies show a difference in threshold of between 4 and 15 dB over the central region as a function of spot size (figure 7 and figure 2 of Khuu and Kalloniatis; Khuu & Kalloniatis, 2015) with threshold being greater for larger spots. By comparing our size scaled targets in the periphery with published data for the GIII stimulus (Figure 7 here), we obtain a difference of 8.3 dB which is consistent with this literature.

Considering the influence of spot size on slope, our findings confirm the hypothesis advanced in the Introduction that the lower contrast threshold in the periphery is accompanied by improved slope (reduced SD of the cumulative gaussian curve fit). As with threshold, the slope was not quite equalized between the central and peripheral points. We further demonstrate broadly similar shapes of the psychometric functions at several eccentricities spanning the range between central and peripheral vision with MOCS data measured using size scaled spots on a tablet perimeter. Unfortunately, this display did not possess sufficient contrast depth to characterize slope near the fovea, but the results nonetheless confirm pronounced lowering of threshold and steepening of slope in the periphery through the use of size scaled stimuli.

It is tempting to suggest that more “aggressive” scaling may be required to completely equalize performance across the visual field. To achieve such scaling, one can decrease the size of macula spots, which makes them more susceptible to refractive error – an undesirable option for clinical applications. Increasing the size of peripheral spots is not an option given that our scaling is already approaching the point of diminishing returns (size V). The reduced variability of scaled spots is predicted to improve the SNR for correct classification of normal points in the periphery. This is consistent with a similar prediction made at a single eccentricity for size-modulated stimuli when testing healthy and glaucomatous individuals (Rountree et al., 2018). However, once the spot reaches a critical size, the benefits for threshold modification cease (or decrease substantially) and the larger spots begin to suffer from poorer spatial resolution of scotoma as evident in the data of Wall et al. (Wall, Doyle, Eden, et al., 2013). As a consequence, we posit that the Goldmann size V spot should be the largest spot used for increment perimetry.

An important caveat to the adoption of larger stimuli for perimetric testing in glaucoma is that reduced variability may be accompanied by an effectively shallower “depth” of defect (Wall, Doyle, Eden, et al., 2013). In other words, the reduced variability in tests obtained from a patient with glaucoma may be offset by the reduced signal, leaving the clinician in no better state to reliably detect a patient with disease. However, we would argue that a large proportion of visual field testing is conducted either on normal subjects, or at least on normal points in the visual field in subjects with mild to moderate glaucoma. Accordingly, the false positive rate is arguably more important in provision of clinical care, and this can be directly addressed by reducing test-retest variability through the use of larger stimuli in the periphery. We note further that the work of Wall et al. compares stimuli of size III and above, whereas the scaling we have advocated here ranges from approximately a size II to a size IV; it is not clear whether the finding regarding more “shallow” defects with large stimuli will hold for these somewhat smaller stimuli.

Another potential reason that our approach did not demonstrate complete “correction” of the hill of vision is that anatomic differences in retinal and cortical sampling are expected across differing retinal locations at the same eccentricity. Such variations may also differ between individuals, especially in the nasal visual field (Swanson, Dul, Horner, & Malinovsky, 2017). Rather than a one-size-fits-all quadrants approach, it may prove beneficial to incorporate region-specific scaling of the size of perimetric stimuli. To verify utility would require a larger number of trials to be delivered within each session and for fixation to be directly monitored, because results could no longer be pooled across quadrants.

In addition to the above considerations regarding detection of pathological change, the Goldmann size V was the largest spot size employed in our study whereby we applied a proportionate increase in size to the stimuli from data reported by Sloan (Sloan, 1961) and constrained so as not to exceed the level of diminishing returns found by others (approximately Goldmann V; Latham et al., 1994; Zele et al., 2006). It is worth noting that the spot sizes considered in our scaling are beyond the critical size for spatial summation at these test locations (Khuu & Kalloniatis, 2015; Wilson, 1970). This implies that these stimuli should test cortical as well as retinal perceptive fields (Pan & Swanson, 2006).

In a clinical test using a limited number of trials, improvements in variability should theoretically provide significant improvements in test-retest repeatability and therefore the ability to make accurate statistical inferences regarding whether a test is normal or abnormal or has progressed compared with a previous test. Our adaptive threshold data collected on the MRF perimeter confirmed low intra-observer variability with size scaled stimuli, which did not appear to vary with eccentricity in contrast to published data collected with the GIII stimulus. Note that our thresholds measured in young subjects are higher (see Figure 7) when compared to a dataset measured in older subjects (Heijl et al., 1987), as might be expected, however, the salient point is the large difference in threshold between central and peripheral test locations. Furthermore, age-related differences are only approximately 1 dB for larger spots (Zele et al., 2006), such that size-scaled peripheral thresholds measured in older eyes should be similar to those reported here. In addition, we note that Heijl et al. did not report any trend for increased test-retest variability with age (Heijl et al., 1987) and that our subjects recruited for the MRF study were perimetrically naïve; therefore, we are hopeful that the strong test-retest repeatability will be replicable on larger cohorts.

In addition to considerations regarding slope and reliability of adaptive threshold testing, using larger spots in the periphery will make the stimuli more robust to blur and lower thresholds thereby extending the dynamic range over which pathological changes may be reliably investigated (Vingrys et al., 2016). Prior work has shown that pathological change resulting in thresholds below about 20 dB is commensurate with a sharp increase in test-retest variability (Gardiner, Swanson, Goren, Mansberger, & Demirel, 2014). Increasing spot size should improve (lower) threshold at such locations; if defect depth is only moderate then performance above the 20 dB “limit” may be restored, allowing defects to be measured more reliably.

## Conclusions

Size scaling of perimetric test spots, to a maximum of Goldmann size V, will increase thresholds and reduce variability at peripheral locations in normal subjects. The increased threshold will give an extended test range at peripheral locations compared with a GIII spot. The reduced variability arises due to improved slope of the psychometric function and should allow better classification of normal points in the periphery.
